# Alpha-glucan: a novel bacterial polysaccharide and its application as a biosorbent for heavy metals

**DOI:** 10.1186/s43141-023-00609-3

**Published:** 2023-11-22

**Authors:** Desouky Abd-El-Haleem

**Affiliations:** Environmental Biotechnology Department, Genetic Engineering and Biotechnology Institute, City of Scientific Research and Technological Applications, Burgelarab, Alexandria, 21934 Egypt

**Keywords:** *Bacillus velezensis*, Exo-polysaccharides, Alpha-glucan, Heavy metals, Removal, Bioremediation, Biosorption, Alginate

## Abstract

This study identified an extracellular bacterial polysaccharide produced by *Bacillus velezensis* strain 40B that contains more than 90% of the monosaccharide glucose as alpha-glucan. A prominent peak at 1074 cm^−1^, a characteristic of glycoside couplings, was visible in the FTIR spectrum. There were traces of xylose, sucrose, and lactose, according to the HPLC study. The ability of this bacterial glucan to operate as a biosorbent of the heavy metals cobalt, chromium, copper, and lead from aqueous solutions was investigated in conjunction with Ca-alginate beads. It proved that glucan 40B has a low affinity for chromium ions and is selective for lead. Initial concentration measurements showed an inverse relationship between concentration and the amount of metal ions eliminated. Lead and chromium removal increased as the glucan dose was increased. It was shown that as the pH of the starting solution is elevated, there is an increase in the sorption of metal ions onto the glucan. It was clear that when the temperature increased, the fraction of metal ion sorption slightly increased. Glucan has a wide range of industrial applications, from food and medicine to health and nutrition. As a result, the investigation’s scope was expanded to include heavy metal removal.

## Background

Heavy metal ions have the potential to dramatically affect both human and animal health because they can attach to proteins, nucleic acids, and small metabolites in living organisms. Heavy metals are concentrated in wastewater, especially from industrial sources, and when they go up the food chain, humans and other animals absorb them. Large amounts can be harmful or dangerous, but small amounts are necessary for good health [1, 2]. The hazardous effects of heavy metals can lower energy levels and impair the health of vital organs like the brain, lungs, kidney, liver, blood, and others [3]. As a result, it is preferable to use low-cost technology to remove heavy metals from wastewater before using it in agriculture or releasing it into any type of water [4]. Heavy metal removal from wastewater by physical–chemical methods such as reverse osmosis, solvent extraction, lime coagulation, ion exchange, and chemical precipitation is expensive and does not provide the required levels of removal. Microbes have recently been recognized as biological adsorbents capable of effectively and cost-effectively extracting heavy metals from wastewater [5]. Various live- and heat-treated fungi were studied to see if they could biosorb heavy metals. The ability for biosorption of heat-treated cells may be greater, equal, or lower than that of their living counterparts [6]. Heavy metal-resistant bacteria, on the other hand, may exist in heavy metal-contaminated locations where resistance and efficacy in heavy metal removal differ markedly between bacteria and fungi [7].

The treatment of wastewater effluents containing heavy metal ions is difficult due to the strong reliance on techno-economic, environmental, and social aspects. It is necessary to employ a variety of measures to reduce water pollution and repair both legacy sites and the aquatic ecosystems that surround them. Due to the complexity of the problem, it is impossible to build a single solution that can handle a variety of wastewater effluents [8]. Chemical precipitation, coagulants, flocculants, membrane filtration, ion exchange, photocatalysis, and adsorption on inorganic materials are examples of currently employed heavy metals removal technologies. The advantages of these traditional technologies are often connected with fast processing times, controllability, resistance to high heavy metal concentrations, ease of operation, and a well-understood molecular basis [9]. Some of them, however, are not sustainable since they depend on nonrenewable resources, such as coal and oil for the production of activated carbon and ion exchange resins. Additionally, they could produce waste byproducts that are challenging to eliminate. They might also consume too much energy [10].

In order to remove heavy metals from liquids, it is crucial to look at low-cost, secure, and long-lasting biomaterials. Several investigations have shown that biosorption is a potent alternative to conventional methods [11]. It is well-liked because of its versatility, affordability, lack of secondary pollutants, low-energy usage, and great efficiency. Given that it employs living things as adsorbents, it might be a realistic choice. Numerous studies have demonstrated that a variety of organisms, including waste from soybean meal, sugarcane bagasse, fungus, and bacteria, may readily absorb heavy metal ions. Though the great majority of research has only evaluated the biosorption capacities of entire microbial cells, a small number has looked into how certain microbial cell components may affect the biosorption process [12].

Glucan and other extracellular polymeric materials can be helpful in the biosorption and bioaccumulation processes. This kind of polymer is reasonably priced, green, and biodegradable [13]. Glucans are polysaccharides present in cereals like oats and barley as well as non-cereal sources such mushrooms, microalgae, bacteria, and seaweed. They have a significant therapeutic benefit since they can be used to treat a range of disorders [14]. This polymer has good sorption properties due to its high concentration of − OH groups, low crystallinity, and well-developed surface. In the present study, in experiments for heavy metal biosorption (cobalt, chromium, copper, and lead), a glucan polysaccharide extracted from the *Bacillus* bacteria strain 40B was identified and used. In the polysaccharide under examination, 90% of the glucose molecules are present.

## Methods

### Glucan 40B purification and characterization

According to Zaki et al. [15], strain 40B was pre-cultured for 24 h on a selective medium containing 1% glucose, 0.35% yeast extract, 0.5% K_2_HPO_4_, 0.2% KH_2_PO_4_, 0.05% MgSO_4_ 7H_2_O, 0.01% NaCl, and 1.5% agar. The broth from this medium (2%) was used as a starter culture for a fermentation medium that contained the following ingredients per liter: FeCl3 40 mg, CaCl_2_ 150 mg, MnSO_4_ 140 mg, NH_4_Cl 7 g, K_2_HPO_4_ 0.5 g, MgSO_4_ 0.5 g, and L-glutamic acid 20 g. The inoculated material was then agitated for 48 h on a rotary shaker at 30 °C and 200 rpm. To purify polysaccharide, the cell-free supernatant of strain 40B was concentrated to 0.2 volumes with a rotary evaporator and dialyzed overnight at 4 °C. Thereafter, three volumes of cold anhydrous ethanol (4 °C) were added to the dialyzed broth. After dissolving the precipitate in deionized water, 10% cetylpyridinium chloride (CPC) was added while stirring. The precipitate was centrifuged at 5000 rpm for 15 min and dissolved in 0.5-M NaCl after several hours. After that, 3 l of cold anhydrous ethanol (4 °C) was added to generate the precipitate. The precipitate was lyophilized after three washes with 75% ethanol to generate pure polysaccharides. According to prior research by Zaki et al. [15], measurements of total sugars, protein content, and composition in terms of sugars and amino acids were made. Its function groups were also assessed using a Shimadzu FTIR-8400 S Fourier transform infrared (FTIR) spectrophotometer with KBr as a matrix and a 4000 to 500 cm^1^ wave number range.

### Heavy metals uptake determination

The biosorption properties of glucan 40B for the removal of heavy metal ions were investigated using four distinct synthetic waste solutions comprising the chloride salts of copper, cobalt, lead, and chromium ions. The analytical reagent grade salts utilized were copper chloride (ACROS, USA), cobalt chloride (Fisher Scientific, UK), lead chloride (Bangalore, India), and chromium chloride (Pratap Chemical Industries Pvt. Ltd., India). Four stock solutions were made by combining known weights (mass were defined based on the grams of solute per 100 ml of solution) of each chloride salt with 200 ml of distilled water. After dissolving, the solution was diluted in double-distilled water to 1000 ml. Ion uptake by glucan 40B was evaluated using Prodigy prism high dispersion inductively coupled plasma-atomic emission spectroscopy with ppb (part per billion) quantitative limits (ICP-AES, USA). Batch mode adsorption studies were carried out using a laboratory flocculator (Flocumatic 6PLAZAS/sample, Spain) to demonstrate the effect of contact time (0–5 h), initial metal ion concentration (1–50 ppm), polysaccharide dosage (0–5 ml), solution pH [1–12], and solution temperature (25–80 °C) on the ion metal biosorption process. Due to its high water solubility (> 99%), glucan 40B was immobilized on alginate and hardened into sphere-shaped beads. It was homogenized at 14,000 rpm for 5 min with a 2% alginate solution. This mixture underwent 10 ml/min peristaltic pumping for 24 h while being gently spun. After that, it was immersed in 3% CaCl2. The beads were then removed, washed with distilled water, and dried at 40 °C for 24 h. The beads were approximately 3 mm in diameter and had a round shape. The true sorption capacity of the biopolymer for heavy metal ions was calculated by subtracting the sorption capacity of alginate spherical beads from the sorption capacity of glucan 40B immobilized beads using the following equation:

$${\mathrm C}_{\mathrm{biopolymer}}={\mathrm C}_{\mathrm{biopolymerbeads}}-{\mathrm C}_{\mathrm{alginatebeads}}$$

where C is the final heavy metal concentration in aqueous solution after phase separation (mg/L). The percentage metal ion sorption and the metal ions uptake capacity using biopolymers were calculated from the following equations, respectively.

$$\%\;\mathrm r\mathrm e\mathrm m\mathrm o\mathrm v\mathrm a\mathrm l=\left(\left(\mathrm{Co}-\mathrm C\right)/\mathrm C\right)\times100$$

$$\mathrm{Q }\left(\mathrm{mg}/\mathrm{g}\right) =\mathrm{ V }\left(\mathrm{Co}-\mathrm{C}\right)/\mathrm{M}$$

## Results and discussion

### Glucan 40B confirmation

**Fig. 1:**
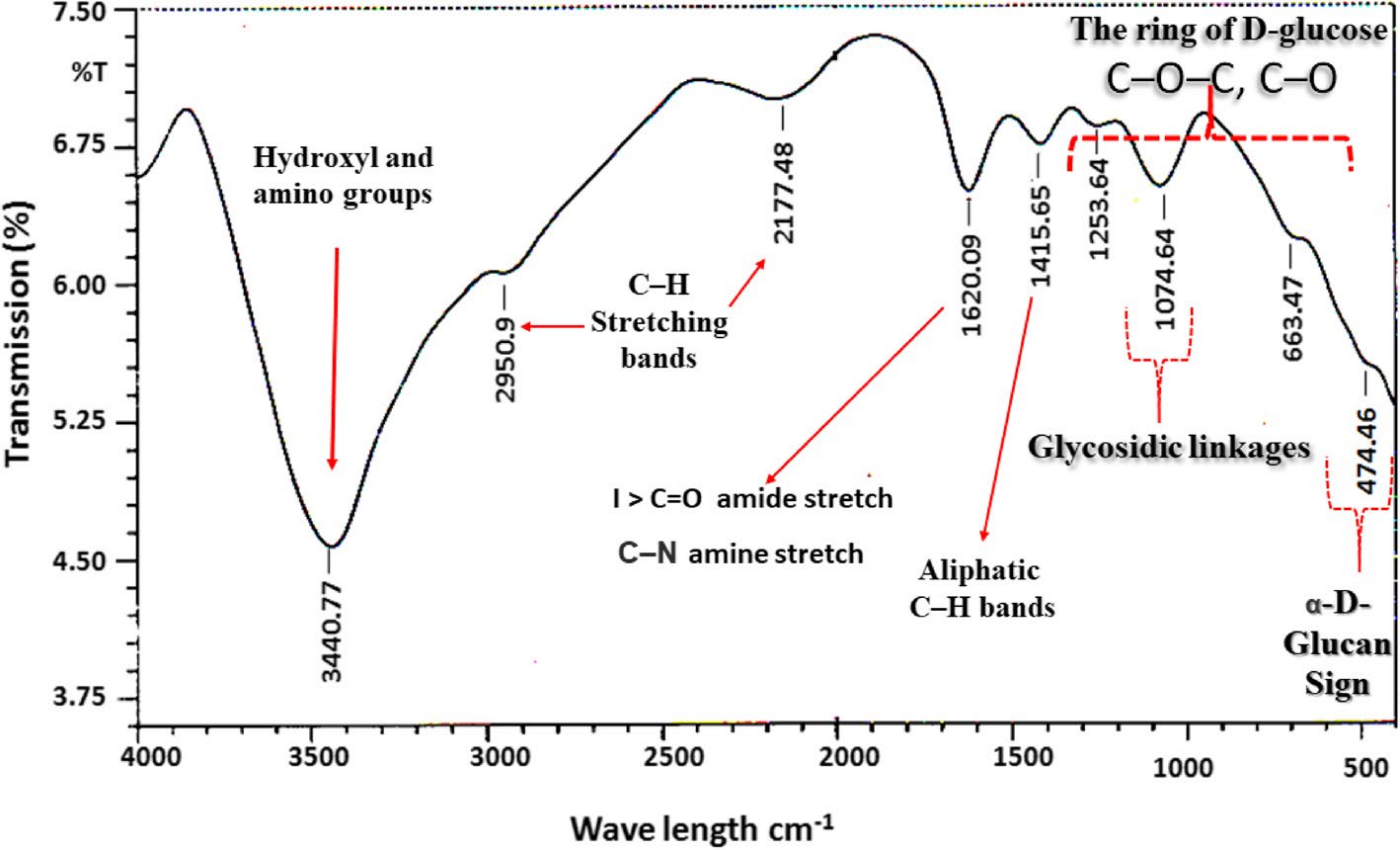
FTIR analysis of the pure bioflocculant 40B reveals the presence of the D-glucose ring as well as the glycoside linkage signature function group of the monosaccharide chain, modified from Zaki et al. [15]

FTIR analysis revealed that the extracellular polysaccharide produced by strain 40B is a glucan (Fig. 1). The existence of a strong broad band about 3440 cm^1^ revealed the presence of O–H stretching in hydrogen bonds, indicating the presence of considerable intra- and intermolecular interactions between polysaccharide chains [16]. The weak band at 2924 cm^1^ is caused by the C–H stretching and bending vibrational modes, according to Kozarski et al. [17]. The C–O–C and C–O bonds of a ring of D-glucose, which are network vibrations in which all of the atoms of the macromolecular chain vibrate in phase and normal modes as a result of coupling of the C–C and C = O stretches, correspond to the region 1285– > 500 cm^1^, which showed peaks with maximum absorption at 1074 cm^1^. The peak at 1074 cm^1^ therefore indicates the presence of glycosidic linkages and cyclic structures in monosaccharides [18]. The novel glucan does not belong to beta-linked glycosyl residues, due to the lack of bands at 900 cm^1^ [19]. Zaki et al. [15], who used HPLC analysis to discover trace amounts of xylose (4.21%), sucrose (2.74%), and lactose (2.34%), previously provided additional confirmations, while glucose made up 90.7% of the novel glucan. After 24 h of incubation, the total biopolymer yield was 3.54 g/L, with 98% sugars and 2% associated protein. Glutamic (40%), aspartic (7.9%), and glycine (6.6%) were the most abundant amino acids. Glucan 40B was soluble in both acidic and alkaline environments, in addition to water.

Polysaccharides, the most prevalent type of carbohydrate found in nature [20], are composed of monosaccharide units linked by glycosidic connections [21, 22]. They can be glycosidically bonded sugar residues or covalently linked compounds such as peptides, amino acids, and lipids. Homoglycans with the same monosaccharides are referred to as homopolysaccharides [21]. These polysaccharides are classified into three structural types: α-glucans, β-glucans, and mixed α, β-D-glucans [4]. For glucan characterization, the arrangement of glycosidic linkages and molecular mass are also relevant. FTIR spectroscopy is a valuable method for polysaccharide structure characterization [23, 24]. The position and anomeric configuration of glycosidic links in glucans are sensitive to this approach. Other molecules, primarily proteins, may be found in the isolated fractions as well. The formation of a conspicuous band at 470–485 cm^−1^ suggested α-D-glucan substitution. According to Zhbankov et al.’s [25] study of monosaccharide and disaccharide model systems, the presence of a volumetric substituent of the OH group at C4 induces the creation of a substantial band in the 470–485 cm^−1^ spectra. According to this interpretation, the current glucan belongs to the α-D-glucan group. A polysaccharide that is found in the mucus produced by many microbes throughout their growth [26, 27].

### Removal of heavy metals

The most popular methods for eliminating heavy metal ions from wastewater include chemical flocculation, oxidation–reduction, adsorption, and electrolysis. Chemical flocculation, one of the most widely used methods, involves the use of chemical reagents that can precipitate out heavy metal ions and produce a material that is insoluble in water [1, 2]. The large secondary contamination that the precipitant will produce is this method’s disadvantage. The adsorption approach, in contrast, is one of the most efficient and environmentally responsible techniques for treating wastewater that contains heavy metals. It is easy to use, has excellent treatment outcomes, is affordable, and does not create secondary pollutants [3, 7, 28]. In the current work, alginates were used to encapsulate glucan 40B in order to provide a cost-effective and long-lasting technique for adsorbing heavy metal ions from industrial effluent. Due to the widespread occurrence of metal-scavenging functionalities, such as carboxyl and hydroxyl, inside the polymer’s backbone chain, alginates, like glucans, are beneficial polysaccharides for the sequestration of heavy metal ions. Additionally, neither polymer emits any secondary pollutants, and they are both biocompatible and degradable [29].

**Fig. 2:**
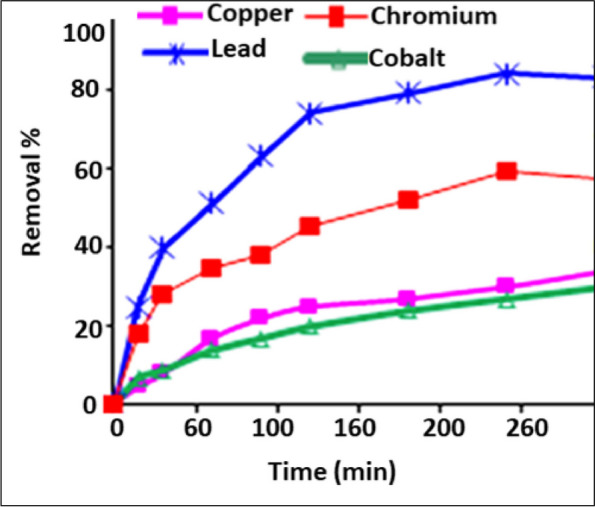
Effect of contact time on heavy metals removal using glucan 40B (volume of waste solution = 100 ml, agitation speed = 250 rpm, dosage of glucan beads = 0.5 g, initial metal ion concentration = 10 ppm, and temperature = 25 °C). The results represent the average of three replicates

**Fig. 3:**
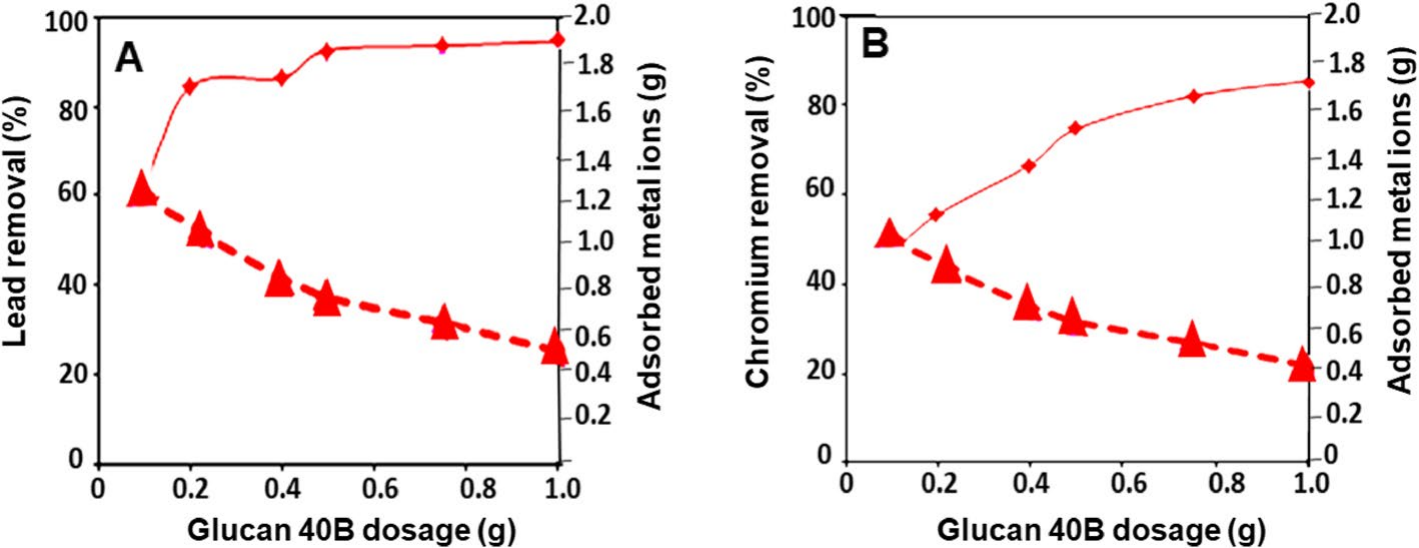
Effect of glucan 40B dose on lead (**A**) and chromium (**B**) metal ions removal capacity and removal (%) (agitation speed: 250 rpm, contact time: 4 h, initial metal ion concentration: 10 ppm, temperature: 25 °C, dosage of glucan beads = 0.5 g, volume of waste solution: 100 ml). The results represent the average of three replicates

The biosorption of copper, cobalt, lead, and chromium onto glucan 40B as a function of time in contact is depicted in Fig. 1. Although glucan 40B has a relative sorption affinity for chromium ions, it is selective for lead ions. After 4 h, metal ion sorption onto glucan began to approach equilibrium, indicating that the sorption process was complete. Figure 2 illustrates how lead removal rose to a point before stabilizing based on the relationship between the amount of glucan, the percentage of metal ions removed, and the amount of metal ions adsorbed (Fig. 2A). On the other hand, increasing the glucan dose improves the removal of chromium ions (Fig. 2B). As a result, the optimal dosage for lead sorption was found to be 5 g of glucan per liter of lead-contaminated solution. Due to an increase in adsorbent surface area and the availability of more metal ion sorption sites, biosorption increases with dosage [30]. However, as the quantity of glucan rose, as depicted in Fig. 3, the unit biosorption for the quantitative removal of metal ions lead (Fig. 3A) and chromium (Fig. 3B) decreased. It was demonstrated that the initial metal ion concentration and the proportion of metal ions removed have an inverse relationship. The creation of metal ion aggregates on the surface of the biopolymer particles may be caused by an increase in metal ion molecules sticking to the outer surface of glucan, which significantly elevates the local metal ion concentration. The amount of biosorption is decreased by these aggregates because they prevent ion transport through the particle.

**Fig. 4:**
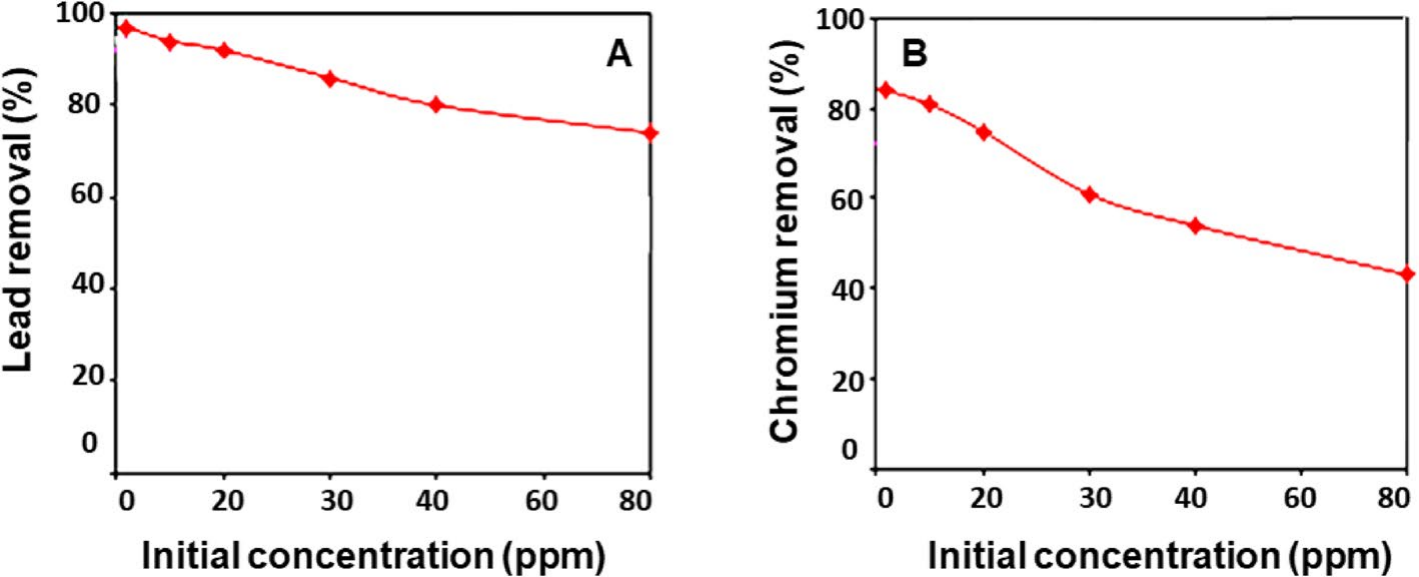
Effect of initial metal ion concentrations on the percentage removal using glucan 40B for the two metal ions: **A** lead and **B** chromium (volume of waste solution = 100 ml, agitation speed = 250 rpm, contact time = 4 h, dosage of glucan beads = 0.5 g, and temperature = 25 °C). The results represent the average of three replicates

**Fig. 5:**
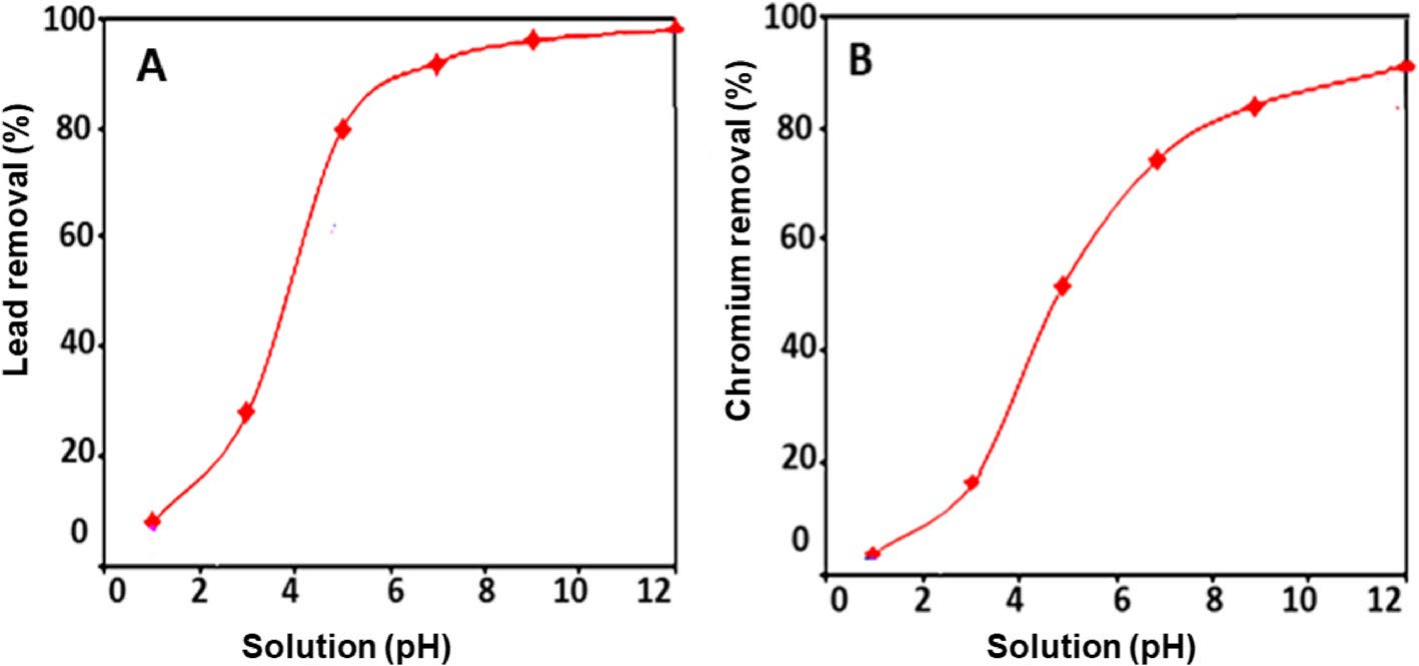
Effect of waste solution pH on the percentage removal using glucan 40B for the two metal ions: **A** lead and **B** chromium (volume of waste solution = 100 ml, agitation speed = 250 rpm, contact time = 4 h, dosage of glucan beads = 0.5 g, initial metal ion concentration = 10 ppm and temperature = 25 °C)

**Fig. 6:**
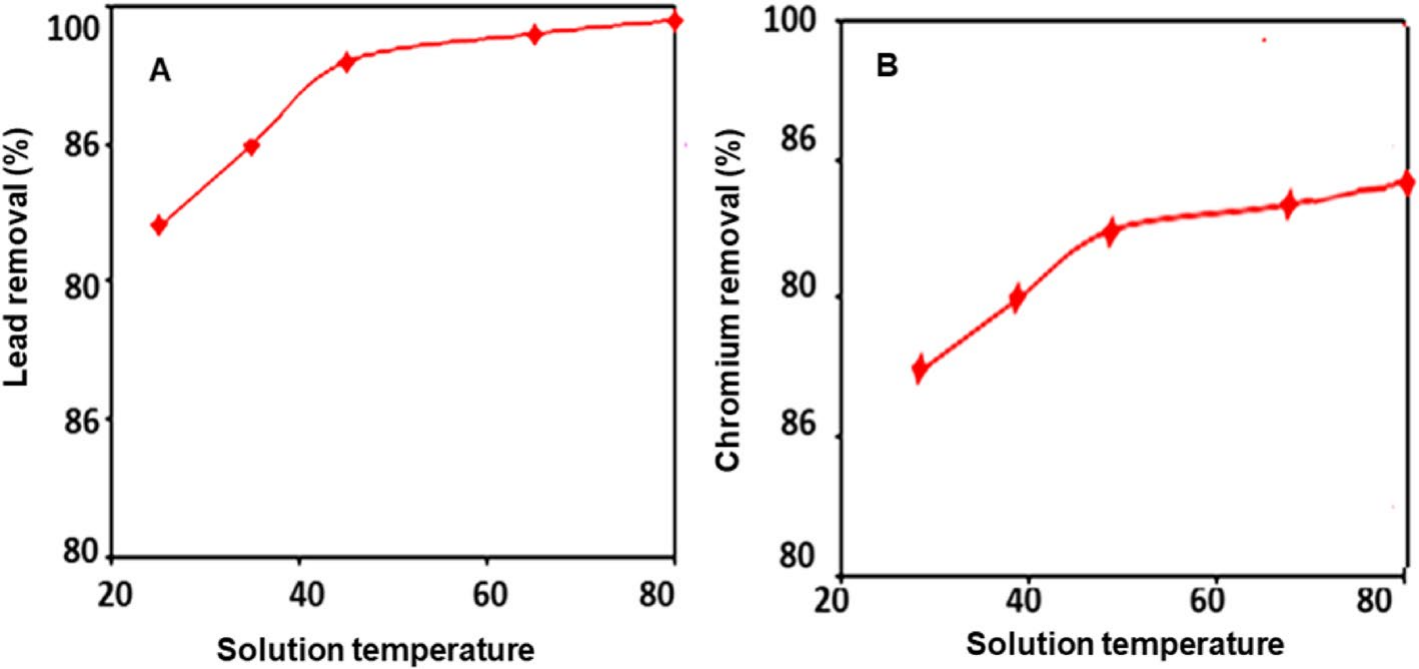
Effect of waste solution temperature on the percentage removal using glucan 40B for the two metal ions: **A** lead and **B** chromium (volume of waste solution = 100 ml, agitation speed = 250 rpm, contact time = 4 h, dosage of glucan beads = 0.5 g, and initial metal ion concentration = 10 ppm). The results represent the average of three replicates

Figure 4 depicts the effect of hydrogen ion concentration on the amount of lead (Fig. 4A) and chromium (Fig. 4B) metal ions that bind to glucan. The sorption of metal ions onto the glucan increases as the pH of the original solution increases. This could be because, at pH levels below 7, a high concentration of H + can compete with metal ions for sorption sites, lowering glucan’s capacity and, as a result, the amount of metal ions removed by it. As the pH of the solution increases (pH > 7), the majority of metal ions tend to precipitate as hydroxides. As a result, rather than metal ion sorption onto the biopolymers, metal ion precipitation is responsible for metal ion removal above pH 7. Figure 5 depicted how the temperature of the solution affected the amount of removed lead (Fig. 5A) and chromium (Fig. 5B) metal ions. This graph clearly illustrates that when the temperature increased, metal ion sorption onto the glucan increased slightly. It is confirmed that the sorption process is almost endothermic by the little temperature advantage in the sorption of metal ions. Higher temperatures may activate the metal ions for enhancing biosorption at the exchanging sites of the biopolymer because cations move more quickly as the temperature rises (Fig. 6).

## Conclusion

This study presents glucan 40B, a novel bacterial polysaccharide classified under the alpha-glucan group, with a wide range of applications in medicine, industry, agriculture, and bioremediation. The objective of this research was to investigate the effectiveness of glucan 40B in removing heavy metals. To improve its usability, glucan 40B was combined with alginate to form dry granules. These granules demonstrated remarkable efficiency in eliminating heavy metals such as chromium, lead, copper, and cobalt from liquid samples. Particularly noteworthy outcomes were observed in the removal of lead and chromium. These findings indicate that glucan 40B shows great potential as a solution for water contaminated with heavy metals. Future studies will involve conducting larger-scale fermentation tests and establishing a semi-industrial pilot plant on our campus.

## Data Availability

The availability of the data is open and free for all public.
